# Utilization of institutional delivery service and associated factors in Bench Maji zone, Southwest Ethiopia: community based, cross sectional study

**DOI:** 10.1186/s12913-017-2057-y

**Published:** 2017-02-01

**Authors:** Niguse Tadele, Tafesse Lamaro

**Affiliations:** Department of Nursing, Mizan Tepi University, College of Medicine and Health Sciences, Mizan Teferi, Ethiopia

**Keywords:** Institutional delivery, Bench Maji, Ethiopia

## Abstract

**Background:**

At the end of Millennium development goals, Ethiopia was included among 10 countries which constitutes about 59% of maternal deaths due to complications of pregnancy and/or childbirth every year globally. Institutional delivery, which is believed to contribute in reduction of maternal mortality is still low. Hence this study was conducted in order to assess utilization of institutional delivery and related factors in Bench Maji zone, Southwest Ethiopia.

**Methods:**

Cross sectional study was employed from September 1st – 30th, 2015 in Bench Maji Zone, Southwest Ethiopia where 765 mothers who deliver 2 years preceding the study provided data for this research. Data were collected by enumerators who were trained. In addition to descriptive statistics, binary and multivariate logistic regression analyses were performed. Statistical significance was considered at a *p*-value < 0.05. Strength of association was also assessed using odds ratios with a 95% confidence intervals.

**Results:**

About 800 mothers were approached but 765 of them who gave birth 2 years preceding the survey participated and gave consent to the data included in the analysis. About 78.30% delivered their last child in health institution while rest gave birth at home. Factors such as maternal age, religion, occupation, availability of information source as TV/Radio, income quartile, residence, knowledge of problems during labor and antenatal follow up had association with institutional delivery which was significant.

**Conclusion:**

In Bench Maji Zone institutional delivery was shown to be comparatively good compared to other studies in the region and in Ethiopia in general even though it is below the health sector transformation plan of Ethiopia which aimed to increase deliveries attended by skilled health personnel to 95%. Empowering women, increasing awareness about institutional delivery and proper scaling up of antenatal care services which is an entry point for institutional delivery are recommended.

**Electronic supplementary material:**

The online version of this article (doi:10.1186/s12913-017-2057-y) contains supplementary material, which is available to authorized users.

## Background

Each year, still at the end of Millennium development goals (MDG), hundreds of thousands of women are dying due to complications of pregnancy and/or childbirth. In 2015, about 99% of the estimated global maternal deaths occurred in developing with Sub - Saharan Africa alone accounting for roughly 66% of deaths [1].

The first hospital based maternal mortality ratio (MMR) estimation in Ethiopia was done in Addis Ababa in 1980 [2]. However till today, Ethiopia is still included among 10 countries which hosts nearly 59% of maternal deaths ranking 4th next to Nigeria, India and Democratic Republic of the Congo with 11,000 (3.6%) maternal deaths [1].

The proportion of maternal death due to different causes changes from year to year. In Ethiopia the main causes of maternal deaths include infection, hemorrhage, obstructed labor, abortion and hypertension in pregnancy [3].

Traditional birth attendants, what ever trained or untrained, are not in a position to predict or get by with solemn complications [4]. Hence; increasing the number of mothers delivering at health facilities with the help of trained professional, which is the main goal of safe motherhood and child survival movements [5, 6] is essential for the betterment of both child and maternal health.

The percentage of institutional deliveries continues to be low in Ethiopia. Nevertheless there has been significant advancement in the past 15 years in which the number of deliveries at health facility is higher, from 5% in 2000 to 10% births reported in 2011 [7].

Home delivery is common in different regions of Ethiopia. For instance, studies have shown that 12.10% of mothers in Sekela district [8], 28.60% in South West Shoa [9], 13.50% in North Gondar [10], 34.21% in Bako district [11], 18.20% in Dodota district [12] and 47% in Goba Woreda [13] gave birth at health facilities. In contrary to this, better utilizations were also documented in different studies with about 61.60% of mothers in Holeta town [14], 71.2% in Southern Tigray [15], 78.8% in Bahir Dar town [16] and 86.5% in Addis Ababa delivering in health institutions [7].

Different studies in Ethiopia showed socio-demographic, obstetric, and health services related factors as determinants for institutional delivery service utilization. Among socio demography related factors residence, access to radio (media exposure), maternal education, wealth, partner and family involvement in decision making on place of delivery, age during first marriage, ethnicity and occupation [9–14, 16] were shown to be significantly associated with delivery at health institution. Regarding obstetric factors parity, experience of a pregnancy and/or delivery related problems, past history of intra partum complication or prolonged labor [9, 10, 12, 14], were reported to be associated. Health service related factors as; time it takes to the health facility, knowledge of the number of recommended ANC visits, good perception about quality of health services given to mother, gestational age during first ANC visit, antenatal care follow up during pregnancy, access to transport, being birth prepared and ready for its complication, perception of distance to health institutions [9–14, 16] were also seen to be associated institutional delivery.

Working towards Sustainable Development Goal (SDG) number 3.1 which aimed to cut down the global MMR below 70 per100, 000 live births and stopping maternal mortality which is preventable by 2030 requires magnifying efforts and advancements catalyzed by MDG 5. Institutional delivery which is believed to contribute to the success of this plan was about 14.9% in Southern Nations Nationalities and Peoples Regional State (SNNPRs) [7] and 38.1% in the current study area (Bench Maji Zone) [17] which is still low.

This study was carried to assess utilization of institutional delivery service and associated factors in Bench Maji zone, Southwest Ethiopia. The finding of this study could help to understand institutional delivery and associated factors and reveals areas that need further study.

Results may give useful insights to health care providers, Health Extension Workers (HEWs) and promoters of community health to realize the situation in the survey area so as to give priority and directing their efforts. Moreover, since there is scarcity of similar study in the survey area, the present study can be used as a bench mark for future research. So that the present study was conducted to assess institutional delivery service utilization and associated factors in Bench Maji zone, Southwest Ethiopia.

## Methods

### Study aim, design, area and period

Community based cross sectional study was taken place in September, 2015 in Bench Maji zone, Southwest Ethiopia to measure institutional delivery service utilization and related factors. Bench Maji zone is one of the 16 zones in SNNPRs located 561 km away from Addis Ababa, the center of Ethiopia, in Southwest direction with an estimated population of 829,493 out of which 418,213 are women, 129,500 are children under five and 26,462 are under 1 year. The expected number of households in the zone is about169,284 and the primary health service coverage of the zone is 92.6% [18] covering a total catchment area of 19965.8 Km2 with majority 86% (1,061,120) of the population living in the rural areas [19]. The zone includes a city administration (Mizan Teferi), 10 Woredas (districts), 246 kebeles (smallest administrative units) (229 rural and 17 urban). There are two Hospitals (Bachuma and Maji Primary) one under construction and one functional (Aman General) Hospital. The zone has about 40 functional health centers and five under construction (Koka, Aroge Birhan, Gabisa, Zozo and Kuju). Additionally, there are 182 functional health posts and 9 under construction, one University and one Health sciences college. The zone has 12 physicians’ and 511 of health professionals of different ranks and 476 health extension workers [19].

### Sample Size determination and sampling procedures

The study participants were selected from child bearing age women that gave birth 2 years preceding the survey and regular residents of the selected Woreda (districts) of the zone. A sample size of 800 was calculated using the Statistical Package OpenEpi, Version 2, open source calculator. Owing to use of multistage cluster sampling, prevalence of women giving birth in the health institutions 61.6% (14) taken from a study area which shares the same population characteristics and provides the largest sample size, error margin of 5%, a 95% confidence level and 10% contingency for non response. Multi stage sampling technique was implemented to identify the study participants. From 10 districts and a city administration, two districts and one city administration were randomly selected. Then three Kebeles from each selected district and city administration were selected randomly using lottery method.

Households which host mothers who delivered 2 years preceding the survey were recorded in a census made by resident data collectors and the allocated sample for selected Kebeles was calculated using probability sampling proportional to the number of households in the Kebele. A total of 800 samples were selected using systematic sampling from the selected nine kebeles. For households with more than one eligible respondent, face-to-face interview was performed by choosing a respondent by lottery method. Two revisits were made when eligible mothers were not available during the visit. Eight female data collectors who had a minimum of Diploma were deployed after 2 day training on the objectives and methods of data collection. The data collection was closely monitored by supervisors and the principal investigator.

### Data processing and analysis

The completed questionnaire was entered to a computer using EpiData version 3.0 and exported to SPSS version 20.0 for analysis. To check for consistency of the data entry frequencies were calculated and 5% of the questionnaire was selected randomly and re entered. Frequency, percentage, chi-square with *p*-value was used for description of the study population in relation to relevant variables. Analysis was performed employing a two step logistic regressions [bivarate and multivariable] since the dependent variable is dichotomous to look for the effect of the independent variables on the dependent variable by controlling confounders. Statistical significance was evaluated at 95% levels of confidence and *p* value less than 0.05 was considered significant.

### Variables in the study

Data were collected on mothers’ age, ethnicity, religion, maternal and husbands level of education and occupation, marital status, average monthly income, availability of communication media such as TV/radio and residence. Obstetric history and decision making related questions like age at first pregnancy, number of deliveries in the last 2 years, previous pregnancy outcomes, husbands desire to have more children and by whom decision was made to decide on the number of children, knowledge about institutional delivery, problems that can occur during pregnancy and child birth and problems that occur during previous pregnancy were also included.

The outcome variable focused on institutional delivery services utilization, by identifying identified mothers who gave birth with the assistance of health professionals trained on skills which are essential to carry off normal delivery, diagnose and refer obstetric complications.

Face-to-face interviews were made to collect data with structured and pre-tested instrument during house to house survey (Additional file 1). Most of questions were prepared in close ended forms including the list of alternatives from different studies including the DHS. An alternative “Other” was used to write responses for each closed ended question. Questions which include age, household income, number of deliveries, number of ANC visits and age at first pregnancy were prepared in open ended form and the responses were recorded and later coded during data analysis. For both open and closed ended questions the list of alternatives was not red to respondents and only clarification of unclear questions to the respondents were made.

## Results

### Socio-demographic profile of respondents

A total of 800 respondents were approached and 765 mothers who deliver 2 years preceding the survey were included in the analysis which yields a non response rate of 4.37%. About 400 (52.2%) of the women were from urban (Zonal Town), 166 (21.7%) semi urban (Woreda Town) and the rest 200 (26.1%) were rural residents. The mean age of respondents was 25.47 ± 4.4 with the Mean average monthly income of 1146.19 ± 1328.1 Ethiopian Birr (ETB) while the average family size was 4 ± 1.5 (ranging from 2 to 11). Majority of mothers were from Bench ethnic group (24.6%) followed by Amhara (20.3%). Majority of mothers were Protestant (48.2%) followed by Orthodox Christian (36.7%). Concerning literacy majority of mothers cannot read and write (40%) with majority of their husband attended primary school (42.5%), about 73.2% of mothers were housewives with 36.3% of their husbands were farmers followed by merchants (34.3%) and majority of families have television (TV)/radio (63.1%) in their house (Table 1).

**Table 1:** Socio demographic characteristics of Mothers in Bench-Maji Zone, South West, Ethiopia, September 2015

Variable	Frequency (%)
Maternal age (Mean 25.47,SD 4.4, R 24)	
15–19	21 (2.7)
20–24	319 (41.7)
25–29	271 (35.4)
30–34	109 (14.2)
> 35	45 (5.9)
Ethnicity *n* = 765	
Bench	188 (24.6)
Amhara	155 (20.3)
Menit	148 (19.3)
Kaffa	140 (18.3)
Other^a^	134 (17.5)
Religion *n* = 765	
Protestant	369 (48.2)
Orthodox	281 (36.7)
Islam	96 (12.5)
Other^b^	19 (2.5)
Maternal Educational level *n* = 765	
Cannot read & write	306 (40.0)
Able to read & write^d^	22 (2.9)
Primary school (1–8)	312 (40.8)
Secondary &above (9–12+)^e^	125 (16.4)
Maternal occupation *n* = 765	
House wife	560 (73.2)
Merchant	75 (9.8)
Farmer	60 (7.8)
Government Employee	44 (5.8)
Other^c^	26 (3.4)
Maternal Marital Status *n* = 765	
Married	724 (94.6)
Single	23 (3.0)
Divorced	15 (2.0)
Widowed	3 (0.4)
Husbands Educational level *n* = 724	
Cannot read & write	165 (22.8)
Able to read & write^d^	51 (7.0)
Primary school (1–8)	308 (42.5)
Secondary & above (9–12+)^e^	200 (27.6)
Husbands occupation *n* = 724	
Farmer	263 (36.3)
Merchant	248 (34.3)
Government Employee	99 (13.7)
Daily laborer	85 (11.7)
Other^c^	29 (4.0)
Availability of TV/Radio =765	
Yes	483 (63.1)
No	282 (36.9)
Income quartiles *n* = 765	
Lowest	194 (25.4)
Middle	204 (26.7)
Upper	367 (48.0)
Residence *n* = 765	
Urban	399 (52.2)
Semi urban	166 (21.7)
Rural	200 (26.1)

Note ^a^Hadiya, Wolayita, Gamo, Tigre, Sheko, Sheka, Yem, Dawero, Dize, Gurage, Oromo, Silte

^b^Traditional religion, Catholic and Apostolic

^c^Private business, Driver, Carpenter, Student, Religious leader, daily laborer

^d^Who haven’t joined any formal education but learned writing and reading informally

^e^Who joined secondary education, technical schools, colleges and university

### Obstetric history of respondents

Three hundred seventy six (49.2%) of mothers gave birth to their first child between the age of 20–24 with the mean age during first child birth of 20.2 ± 2.75. Teenage pregnancy (15–19) which have association with high morbidity and mortality for both mother and child was also significantly higher among participants (43.7%). Among the participants 3.7% have delivered children less than a year apart. Concerning the outcomes of pregnancy, 10 (1.31%) of mothers never gave live birth, 4.9 and 4.0% of mothers also have a history of still birth and any forms of abortion. Majority of respondents (67.1%) jointly decided the number of children they have (Table 2).

**Table 2:** Obstetric history of the respondents in Bench-Maji Zone, South West, Ethiopia, September 2015

Variable	Frequency (%)
Age at first pregnancy *n* = 765	
15–19	334 (43.7)
20–24	376 (49.2)
25–34	55 (7.2)
No of deliveries in the last 2 Years *n* = 765	
1	737 (96.3)
2	28 (3.7)
Husband want to have more children *n* = 724	
Yes	489 (67.54)
No	87 (12.02)
Don’t Know	148 (20.44)
Responsible for deciding to have children *n* = 765	
Wife/me	126 (16.5)
Husband	125 (16.3)
Joint discussion	513 (67.1)
Family	1 (0.1)
Pregnancy outcomes in life time	
All live birth	755 (98.69)
History of Still birth	37 (4.9)
History of any type of Abortion^a^	31 (4.0)

^a^Includes spontaneous or induced type of abortion

### Knowledge of danger signs of pregnancy

**Fig. 1:**
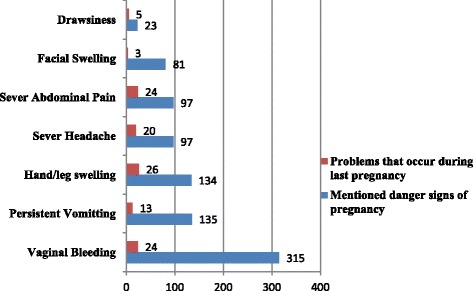
Mentioned danger signs of pregnancy and problems that occur during last pregnancy Bench Maji Zone, Ethiopia, 2015

Three hundred eighty eight (50.7%) of mothers replied that they can identify danger signs that may indicates problems that occur during pregnancy, of whom about 81.19% mentioned vaginal bleeding and only 5.93% mentioned drowsiness as a danger sign of pregnancy Among all mothers, 14.9% replied they had a problem during last pregnancy where hand/leg swelling occurred in about 22.81% of mothers (Fig. 1).

### Institutional delivery related knowledge and service utilization

Majority of respondents (95.6%) replied that they have heard about institutional delivery. Health institutions and health care providers were sources of information for majority (68.9%) of the respondents. About 81.0% replied that they know a health problem that can occur during childbirth in which severe bleeding was mentioned by majority (71.8%) of respondents.

Among mothers who were asked about their place of delivery, majority (78.30%) delivered their last child in health institution and about 21.70% delivered at home. The main reason mentioned for institutional delivery was that “insisted by health professionals and others” to deliver in health institution (82.1%). and the widely mentioned reason for home delivery was “the labor was going well” (62.9%) and some of mothers were also forced to deliver at home since the labor started suddenly. Concerning the final decision on place of delivery, majority (61.70%) replied by joint discussion with husband and about one third (34.20%) replied they decided on their own (Table 3).

**Table 3:** Institutional delivery Service Utilization in Bench-Maji Zone, South West, Ethiopia, September 2015

	Frequency (%)
Ever heard about institutional delivery (ID) *n* = 765	
Yes	731 (95.6)
No	34 (4.4)
Source of information about ID *n* = 731	
Health institution/care provider	504 (68.9)
Family/Relatives	75 (10.3)
Radio/TV	128 (17.5)
Other^a^	24 (3.3)
Know a health problem that can occur during childbirth *n* = 731	
Yes	592 (81.0)
No	139 (19.0)
Mentioned Problems that can occur during childbirth *n* = 592	
Severe bleeding	425 (71.8)
Obstructed labor	219 (37.0)
Fetal death	305 (51.5)
Maternal death	247 (41.7)
Other^b^	14 (2.36)
Place of delivery of the last child *n* = 765	
Institution	599 (78.30)
Home	166 (21.70)
Why they prefer to ID *n* = 599	
Was informed to deliver in HF	492 (82.1)
Faced obstetric problems which forced them to deliver in HF	56 (9.3)
Because previous bad experience	27 (4.5)
Other^c^	73 (12.19)
Why they prefer to deliver home *n* = 132	
The labour was going well	83 (62.9)
I feel more comfortable at home	11 (8.3)
It is my usual practice	29 (22.0)
Other^d^	14 (10.61)
Problem that occur during last delivery *n* = 731	
No problem faced	695 (95.08)
Some problem faced	36 (4.92)

^a^From School, didn’t identify the source

^b^Anemia, Breach presentation, retained placenta, child suffocation

^c^It is better than home, my usual practice, labor was prolonged at home, was sick that time, better for me and my child safety, husband enforced me, nobody at home to care of me

^d^Close attention from relatives & family numbers, No transportation services, Cannot pay for transportation services, my husband’s decision, the labor was suddenly started

### Factors associated with institutional delivery service utilization

Different factors have been identified to have a positive or negative association with utilization of institutional delivery service. On bivariate analysis of the selected factors with institutional delivery; age of the mother, religion, maternal education, maternal occupation, availability of TV/radio, income quartiles, residence, knowledge of problems that may occur during labor, ANC follow up were found to be significantly associated. After adjustment of confounding factors using multivariable logistic regression analysis maternal education lost its association with institutional delivery (Table 4).

**Table 4:** Factors associated with institutional delivery service utilization in Bench - Maji Zone, Ethiopia, September 2015

	Place of delivery							
	Institution		Home					
Variables	N	(%)	N	(%)	COR (95% CI)	*P*-value	AOR (95% CI)	*P*-value
Maternal age								
15–24	305	50.9	35	21.1	1		1	
25–34	269	44.9	111	66.9	0.3 (0.2,0.4)	0.000*	0.4 (0.2,0.7)	0.003*
35–44	25	4.2	20	12.0	0.1 (0.1,0.3)	0.000*	0.3 (0.1,0.8)	0.013*
Religion								
Protestant	246	41.1	123	74.1	1		1	
Orthodox	255	42.6	26	15.7	4.9 (3.1,7.8)	0.000*	2.2 (1.1,4.5)	0.02*
Islam	92	15.4	4	2.4	11.5 (4.1,32.0)	0.000*	1.3 (0.4,4.6)	0.684
Other ^a^	6	1.0	13	7.8	0.2 (0.1,0.6)	0.004*	0.5 (0.1,1.6)	0.236
Maternal educational level								
Can’t read	187	31.2	119	71.7	1		1	
< =Primary	292	48.7	42	25.3	4.4 (3.0,6.6)	0.000*	1.0 (0.5,2.0)	0.939
> =Secondary	120	20.0	5	3.0	15.3 (6.1,38.5)	0.000*	2.1 (0.5,8.8)	0.289
Maternal occupation								
House wife	435	72.6	125	75.3	1		1	
Government	38	6.3	6	3.6	1.8 (.8,4.4)	0.184	0.9 (.2,3.7)	0.864
Merchant	71	11.9	4	2.4	5.1 (1.8,14.2)	0.002*	12.3 (1.6,94.1)	0.015*
Farmer	33	5.5	27	16.3	0.4 (.2,.6)	0.000*	1.8 (.8,4.1)	0.142
Other ^b^	22	3.7	4	2.4	1.6 (.5,4.7)	0.408	0.3 (.1,1.2)	0.098
Maternal marital status								
Married	565	94.3	159	95.8	1			
Other	34	5.7	7	4.2	1.4 (0.6,3.1)	0.462		
Availability of TV/Radio								
No	167	27.9	115	69.3	1			1
Yes	432	72.1	51	30.7	5.8 (4.0,8.5)	0.000*	2.7 (1.4,4.8)	0.002*
Income quartile								
Lowest	117	19.5	77	46.4	1		1	
Middle	139	23.2	65	39.2	1.4 (.9,2.1)	0.104	0.5 (0.3,0.9)	0.033*
Upper	343	57.3	24	14.5	9.4 (5.7,15.6)	0.000*	0.2 (0.1,0.4)	0.000*
Residence								
Urban	382	63.8	17	10.2	1		1	
Semi urban	117	19.5	49	29.5	0.1 (0.1,0.2)	0.000*	0.1 (0.02,0.2)	0.000*
Rural	100	16.7	100	60.2	0.04 (0.03,0.1)	0.000*	0.03 (0.01,0.1)	0.000*
Maternal age during first pregnancy								
15–19	257	42.9	77	46.4	1			
20–24	305	50.9	71	42.8	1.3 (.9,1.9)	0.173		
25–34	37	6.2	18	10.8	0.6 (.3,1.1)	0.124		
Knowledge of problems during labor								
No	104	17.4	69	41.6	1		1	
Yes	495	82.6	97	58.4	3.4 (2.3,4.9)	0.000*	11.0 (5.2,23.1)	0.000*
ANC follow up								
None	25	4.2	57	34.3	1		1	
< 4	147	24.5	88	53.0	3.8 (2.2,6.5)	0.000*	7.6 (3.5,16.8)	0.000*
> =4	427	71.3	21	12.7	46.4 (24.4,88.2)	0.000*	26.2 (11.2,61.2)	0.000*

*N* number/frequency

*Significant association

^a^Traditional religion, Catholic, Hawariyat

^b^Private business, Driver, Carpenter, Student, Religious leader, daily laborer

This study found that mothers in the age groups of 25–34 and 35–44 were less likely to deliver in health institution (AOR = 0.4 and 0.3) respectively in comparison with those mothers in the age group of 15–24. Mothers who are followers of Orthodox Christianity were about two times more likely to deliver in health institution (AOR = 2.2) in comparison with Protestants while Merchants were about 12 times more likely to deliver in health institution (AOR =12.3) in comparison with House wives.

Availability of information source as TV/Radio had shown a significant association in which those mothers who replied they have TV/radio at home were more likely to deliver in health institution (AOR = 2.6) in comparison with those mothers who replied they don’t have TV/Radio at home. Concerning their income, in comparison with those mother’s in the lowest income quartile, those in the middle (AOR = 0.5) and upper income quartile (AOR = 0.2) were less likely to deliver in health institution. In comparison with mothers residing in urban areas, mothers from semi urban (AOR = 0.1) and rural areas (AOR = 0.03) were less likely to deliver in health institution.

Knowledge of problems during labor also had a significant association with place of delivery. Mothers who said they know problems which may occur during labor were 11 times more likely to deliver in health institution (AOR = 11.0) in comparison to those mothers who replied they don’t know problems during labor and in comparison to mothers who had no ANC follow up at all, those mothers who had ANC visits (i.e. less than the recommended four visit or within recommended four, and above visits) were more likely to deliver in health institution (AOR =7.6 and 26.2) respectively (Table 4).

## Discussion

This study assessed utilization of institutional delivery service and associated factors among mothers who delivered 2 years preceding to the survey in Bench Maji zone. Among respondents, majority (78.30%) delivered their last child in health facility. This result was higher than studies done in different study areas including the previous study which was conducted in the present study area (38.1%) [17], the national DHS study in which 16% of deliveries in Ethiopia and 14.9% of deliveries in SNNPR were at a health facility [7] and studies done in Sekela district (12.1%), North Gondar (13.5%), Bako (34.21%), Dodota district (18.2%) and Goba Woreda (47%) where mothers delivered their last child in health institutions [8, 10–13].

This registered high service utilization in this study area might be attributed to the wider introduction and increased number of HEWs in Ethiopia in which more than 38,000 HEWs have been trained and deployed in agrarian, pastoralist and urban areas in more than 16,000 Kebeles [20] which improves the utilization of health services by linking community and health facilities particularly health centers.

Additionally this study was conducted after the government made delivery services to be free at all levels of health institutions and implementation of Health Development Army (HDA) has launched in 2011 with development being made in the organization and network formation over the past 3 years. According to Ministry of Health’s (MOH) annual report, a total of 442,773 HDA groups with 2,289,741 one-to five networks were formed in 2014 [20].

The other possible reason for increased service utilization may be attributed to improved Health infrastructure as the government provided special concern for capacity building and included in Health Sector Development Plan (HSDP) IV strategic objective capacity building to improve health infrastructure [21] and in the present study area during the data collection period there was one Hospital, five Health centers and nine health posts under construction [18]. This was also indicated on a national service provision assessment in which almost all (99%) of government managed facilities offer normal delivery services [22].

High level political commitment and the involvement of Non Governmental Organizations and provision of ambulances on top of expansion of health facilities and deployment of midwives and the emphasis given towards encouraging all mothers to gave birth at health institutions (with the general motto of having “Home delivery free Kebeles”) may be among the reasons for the observed increased number of mothers who gave birth at health institutions. Moreover; in comparison with the time other studies were carried, currently there is increased access of information, education and communication (IEC) on maternity service and improving the status of women in the community which may be the reasons for high coverage. Additionally some of researches were conducted on rural communities [10] unlike the present study which was conducted both on urban and rural. The findings of this study also yield comparable result with other studies in Ethiopia where 61.6% of mothers in Holeta [14], 71.2% in Southern Tigray [15], 78.8% in Bahir Dar town [16] and 86.5% Addis Ababa [7] delivered in health institutions.

In this study maternal age, religion, maternal occupation, availability of information source as TV/radio, income, residence, knowledge of problems during labor and ANC follow had statistically significant associations independently with institutional delivery which is similar with other studies such as South West Shoa in which institutional delivery was positively associated with urban residence, wealth, knowledge of the recommended number of ANC visits and birth preparedness [9]. Results also supported as a study done in North Gondar which found that mothers who have no access to radio were less likely to deliver at a health facility than women with access to radio, rural women were less likely to use the services than urban women, mother with lower incomes (<100 ETB) were less likely to deliver at health institution than those having incomes of 500 Birr and above [10]. Results from this study were also similar with a study done in Bako, Oromia where being a house wife, private employee, ANC visit were associated with institutional delivery independently [11]. Similarly, this study found similar results with a study conducted in Dodota which found that mothers residing in urban set up were more likely to deliver at health institution in comparison with rural and semi urban women [12].

Similar with this study, age of the mother was shown to be associated with institutional delivery in studies conducted in North Gondar [10] and Sekela district [8] which might possibly younger women are educated. Older mother might also consider delivering at home by considering it is not risky since they might have delivered at home previously.

## Conclusions

Utilization of institutional delivery services in Bench Maji Zone was shown to be comparatively good (78.3%) compared to other studies in the region and in Ethiopia even though it is below the HSTP of Ethiopia to increase deliveries attended by skilled health personnel to 95% [20].

Health professionals’ contribution in awareness creation and increased service utilization was also acknowledgeable. To save maternal, newborn and child lives improving the quality of the existing services and enhancing access to withdraw the inequalities in Maternal and Child Health service is of major importance.

Maternal occupation and income have shown relationship with outcome variables. Hence empowering women in the aspect of economy, promoting maternal independence within the family, empowering them to earn and control over the utilization of services and decide on their own health. Relationships between presence of sources of information like TV/radio implies provision of adequate IEC and increasing awareness about institutional delivery through mass media and religious leaders and health professional by giving due emphasis to rural mothers since may have limited access to information than urban mothers.

Knowledge of problems during labor and ANC follow up has shown significant association with institutional delivery. Therefore, proper scaling up of ANC service; which is an entry point for other MCH services quality and follow up with special attention on counseling on problems that may occur during labor and safe institutional delivery. To enhance quality and avoid the inequalities of services the efforts by MCH directorate of the Federal MOH should be expanded in a sustainable way. Regional health bureaus should increase their partnership with higher educational institutions, ministry offices, professional associations and local and international partners and continue awarness creation activities for MCH services through HDA and HEWs by strengthening the logistics management system, appropriate organization and capacity building of HDA since the function of HDA in developing regions and urban areas has not matured yet.

## Additional file

Additional file 1: Questionnaire on assessment of Institutional delivery service utilization and associated factors in Bench Maji zone, Southwest Ethiopia: a community based cross sectional study. This questionnaire was prepared in order to assess institutional delivery service utilization among women who found in Bench Maji Zone. The questionnaire was adapted from the DHS and other literatures and is organized in to three sections namely; Socio-demographic characteristics, obstetric history and danger signs and knowledge and utilization of institutional delivery services. (DOCX 16 kb)

## Abbreviations

ANC: Antenatal care
AOR: Adjusted odds ratio
CI: Confidence interval
DHS: Demographic and Health Survey
EDHS: Ethiopian Demographic and Health Survey
EMDHS: Ethiopian Mini Demographic and Health Survey
ESPA+: Ethiopian Service Provision Assessment Plus
ETB: Ethiopian Birr
HDA: Health Development Army
HEW: Health Extension Workers
HSDP: Health Sector Development Plan
HSTP: Health Sector Transformation Plan
IEC: Information, Education and Communication
MCH: Maternal and child health
MDG: Millennium development goals
MMR: Maternal mortality
OR: Odds ratio
SBA: Skilled birth attendant
SDG: Rate sustainable development goal
SNNPRs: Southern Nations Nationalities and Peoples Regional State
TV: Television

## References

[1] WHO, UNICEF, UNFPA, World Bank Group and the United Nations Population Division (2015). Trends in maternal mortality: 1990 to 2015.

[2] Frost O (1984). Maternal and perinatal death in an Addis Ababa hospital. Ethiop Med J.

[3] Abdella A (2010). Maternal mortality trend in Ethiopia. Ethiop J Health Dev.

[4] United Nations Economic Commission for Africa, African Union, African Development Bank Group and UNDP (2013). Assessing Progress in Africa towards the Millennium Development Goals; Food security in Africa: Issues, challenges and lessons.

[5] Stephenson R, Baschieri A, Clements S, Hennink M, Madise N (2006). Contextual influences on the use of health facilities for child birth in Africa. Am J Public Health.

[6] Kesterton A, Cleland J, Sloggett A, Ronsmans C (2010). Institutional delivery in rural India: the relative importance of accessibility and economic status. BMC Pregnancy Child Birth.

[7] Central Statistical Agency [Ethiopia] (2014). Ethiopia Mini Demographic and Health Survey 2014.

[8] Shimeka A, Mazengia F, Meseret S (2012). Institutional delivery service utilization and associated factors among mothers who gave birth in the last 12 months in Sekela District, North West of Ethiopia: a community - based cross sectional study. BMC Pregnancy Childbirth.

[9] Wilunda C (2015). Determinants of utilization of antenatal care and skilled birth attendant at delivery in South West Shoa Zone, Ethiopia: a cross sectional study. BMC Reproductive Health.

[10] Nigussie M, Hailemariam D, Mitike G (2004). Assessment of safe delivery service utilization among women of childbearing age in north Gondar Zone, north west Ethiopia. Ethiop J Health Dev.

[11] Ejeta E, Niguse T (2015). Determinants of Skilled Institutional Delivery Service Utilization among Women Who Gave Birth in the Last 12 Months in Bako District, Oromia, Ethiopia, 2012/13 (Case–control Study Design). J Gynecol Obstet.

[12] Fikre A, Demissie M (2012). Prevalence of institutional delivery and associated factors in Dodota Woreda (district), Oromia regional state, Ethiopia. BMC Reproductive Health.

[13] Bogale D, Markos D (2014). Institutional delivery service utilization and associated factors among child bearing age women in Goba Woreda, Ethiopia. J Gynecol Obstet.

[14] Birmeta K, Dibaba Y, Woldeyohannes D (2013). Determinants of maternal health care utilization in Holeta town, central Ethiopia. BMC Health Serv Res.

[15] Bayu H, Fisseha G, Mulat A, Yitayih G, Wolday M (2015). Missed opportunities for institutional delivery and associated factors among urban resident pregnant women in South Tigray Zone, Ethiopia: a community-based follow-up study. Glob Health Action.

[16] Abeje G, Azage M, Setegn T (2014). Factors associated with Institutional delivery service utilization among mothers in Bahir Dar City administration, Amhara region: a community based cross sectional study. BMC Reproductive Health.

[17] Abamecha F, Tesfaye T (2016). Delivery site preferences and associated factors among married women of child bearing Age in Bench Maji Zone, Ethiopia. Ethiopian J Health Sciences.

[18] Bench Maji Zone (2015). Adjusted Population numbers in different categories for health care planning. Presented on 2007 EC/2015 Health Sector annual review meeting.

[19] FDRE CSA. National population and housing census of 2007. Addis Ababa: Central Statistical Agency(CSA) of Ethiopia; 2008.

[20] FDRE Ministry of Health (2015). Health Sector Transformation Plan(HSTP) 2015/2016 - 2019/2020. Five-year national health sector strategic plan.

[21] FDRE Ministry of Health (2010). Health sector development program IV 2010/11 – 2014/15. Five year plan.

[22] Ethiopian Public Health Institute, FMOH, ICF (2014). Ethiopia service provision assessment plus survey(ESPA+). Key findings.

