# Supplementary material for: Utilization of institutional delivery service and associated factors in Bench Maji zone, Southwest Ethiopia: community based, cross sectional study
Source: BMC Health Serv Res. 2017 Feb 1;17:101. doi: 10.1186/s12913-017-2057-y (PMC5286839; doi:10.1186/s12913-017-2057-y)
Supplement: Additional file 1: — Questionnaire on assessment of Institutional delivery service utilization and associated factors in Bench Maji zone, Southwest Ethiopia: a community based cross sectional study. This questionnaire was prepared in order to assess institutional delivery service utilization among women who found in Bench Maji Zone. The questionnaire was adapted from the DHS and other literatures and is organized in to three sections namely; Socio-demographic characteristics, obstetric history and danger signs and knowledge and utilization of institutional delivery services. (DOCX 16 kb) [file 12913_2017_2057_MOESM1_ESM.docx]

##

**Mizan Tepi University**

**College of Medicine and Health Sciences**

Questionnaire on assessment of Institutional delivery service utilization and associated factors in Bench Maji zone, Southwest Ethiopia: a community based cross sectional study.

**Consent form that certify the respondents agreement before the interview**

1. Name of the Kebeles __________________________
2. Questionnaire Identification Number_______________________

**Introduction**

Good morning, Good afternoon [According to its convenience]. My name is ________________. I am Nurse/Midwife professionally and now I am collecting data from pregnant women of reproductive age groups(15- 49 years) for the research being conducted to identify factors affecting utilization of institutional delivery service utilization and associated factors in Bench Maji zone, Southwest Ethiopia: a community based cross sectional study.by Mr. Tafesse Lamaro and Mr. Niguse Tadele from Mizan Tepi University, College of Medicine and Health sciences. You are selected to be one of the participants in the study by chance. The study will be conducted through interview. Your name and other personal identifiers will not be recorded on data collection format and the information that you give us will be kept confidential and will also be used for this study purpose alone. A code number will identify every participant and no names will be used. If a report of the result is published, only summarized information of the total group will appear. The interview takes 30 minutes on average and is voluntary and you have the right to participate, or not to participate or to refuse at any time during the interview. You will not face any problem if you do not agree to the information to be asked . Your participation on this study helps to improve and identify factors affecting institutional delivery service utilization in Bench Maji zone. If you have any questions about this study you can ask me or contact the principal investigators Mr. Tafesse Lamaro (Mobile: +251912922271or E-mail: [lamaro.tafesse@yahoo.com](mailto:lamaro.tafesse@yahoo.com) ) or Mr. Niguse Tadele (Mobile: +251913163130 or E-mail: [niguse99@gmail.com](mailto:niguse99@gmail.com))

Are you willing to participate in the study?

1. Yes 2. No

- Interviewer who certified that the informed consent has been given verbally from the respondents

Name_____________________________ Signature__________________

Date______________________________

- Result

1. Completed
2. Refused
3. Partially completed

- Checked by:

Name ______________________ signature_________ Date__________

**Instruction**: For the questions that have alternatives, encircle to the response of the mother. Write appropriate response(s) on the space provided for questions for which alternatives are not given.

**Part I: Socio-Demographic Characteristics of Respondents**

| **S/N** | **Questions** | **Responses** | **Remark** |
| --- | --- | --- | --- |
| 101 | What is your Age? | _________Years |  |
| 102 | What is your Ethnicity? | Bench------------------------------------0  Menit -----------------------------------1  Amhara----------------------------------3  Other(Specify) ----------------------99 |  |
| 103 | What is your Religion? | Protestant-------------------------------0  Orthodox--------------------------------1  Muslim-----------------------------------2  Catholic-----------------------------------3  Other (Specify) --------------------------99 |  |
| 104 | Maternal Educational level? | Cannot read and write ---------------0  Able to read and write ----------------1  Primary school (1- 8) --------------2  Secondary school (10- 12) --------------3  College diploma and above ------------------4 |  |
| 105 | What is your occupation?(Maternal); | House wife---------------------0  Government Employed.------------------1  Daily laborer----------------------2  Merchant ---------------------3  Farmer---------------------4  Others(specify)--------------------99 |  |
| 106 | What is your Marital status? | Single---------------0  Married --------------1  Divorced--------------2  Widowed--------------3  Others(specify) ------------99 | If 1 (Married) ask Q 107 & 108 if not skip to 109 |
| 107 | Educational status of your husband? | Cannot read and write ---------------0  Able to read and write ----------------1  Primary school (1- 8) --------------2  Secondary school 10- 12) --------------3  College diploma and above ------------------4 |  |
| 108 | What is your partner’s occupation? (For Married only) | Farmer -----------------------------0  Government Employee---------------1  Daily laborer----------------------2  Merchant ----------------------------3  Others[specify] --------------------------99 |  |
| 109 | Estimated household monthly income? | --------------------------ETB |  |
| 110 | Residence? | Urban ------------------0  Semi urban---------------1  Rural----------------------2 |  |
| 111 | Do you have radio/TV in your home? | Yes-----------0  No ----------1 |  |

**Part II: Obstetric History and danger signs of pregnancy**

| **S/N** | **Questions** | **Responses** | **Remark** |
| --- | --- | --- | --- |
| 201 | What was your age at your first pregnancy? | ______________ years |  |
| 202 | How many pregnancies have you ever had? (Including any type of Abortion and still birth) | Number of Pregnancy________  Number of Abortion_________  Number of live birth_______  Number of still birth________ |  |
| 203 | How many deliveries have you had in the last 2 years? | ___________ deliveries |  |
| 204 | How long was your last delivery? | __________months |  |
| 205 | Would you like to have more children in the future? | Yes ------------------0  No -------------------1 |  |
| 206 | Do your husband/ partner want to have more children in the future? | Yes ------------------0  No ------------------1  Don’t know ---------------2 |  |
| 207 | Who is responsible for deciding to have children in your family? | Wife/me----------------------------0  Husband ---------------------------1  Joint discussion-------------------2  Other (specify) ------------------99 |  |
| 208 | Do you know danger signs of health problems related to pregnancy? | Yes ------------------0  No ------------------1 | **If no skip to Q 301** |
| 209 | If yes for Q211, can you mention some of the danger signs of health problems related to pregnancy? (**More than one answer is possible**) | Vaginal bleeding -----------------0  Severe Headache----------------1  Severe abdominal pain ---------2  Drowsiness -----------------------3  Facial swelling--------------------4  Hand/leg swelling ---------------5  Persistent vomiting---------------6  Other(specify)-----------------99 |  |
| 210 | Have you had any health related problems during last pregnancy? | Yes ------------------0  No ------------------1 | **If no skip to Q 301** |
| 211 | If yes for Q212, can you mention some of the health related problems during last pregnancy? (**More than one answer is possible**) | Vaginal bleeding -----------------0  Severe Headache-----------------1  Severe abdominal pain ---------2  Drowsiness -----------------------3  Facial swelling-------------------4  Hand/leg swelling ---------------5  Persistent vomiting--------------6  Other (specify)-----------------99 |  |

**Part III: Knowledge and practice on institutional delivery services**

| **S.N** | **Questions** | **Responses** | **Remark** |
| --- | --- | --- | --- |
| 301 | Have you ever heard about institutional delivery service? | No ------------------0  Yes ------------------1 | **If “No” thank them and finish** |
| 302 | Where do you hear about institutional delivery services? | Health institution/ care provider -------------------------------------0  Family/Relatives -----------1  Radio/TV-------------------2  Other(Specify)-------------99 |  |
| 303 | Do you know a health problem that can occur during childbirth? | No ------------------0  Yes------------------1 | **If “No” skip to Q 305** |
| 304 | If “yes” to **Q403**, which of the following problems do you know? **(Multiple answers are possible)** | Severe bleeding------------0  Obstructed labour------------1  Fetal death---------------2  Maternal death---------------3  Others[specify] -------------99 |  |
| 305 | Where did you deliver your last child? | At home -------------------0  In health facilities-----------1 | **If at home skip to Q 307** |
| 306 | If your response to **Q305**  is “at health facility”, Why you preferred to deliver in health facility? **(Multiple answers are possible)** | Because of my previous bad experience from home delivery --------------------------------------0  I was informed to deliver in health Facilities------------------1  I have faced obstetric problems which forced me to deliver in health facility---------------------2  Others(specify) -------------99 |  |
| 307 | Why you preferred home delivery? **(Multiple answers are possible)** | The labour was going well------0  I feel more comfortable at home --------------------------------------1  Close attention from relatives & family numbers-------------------2  It is my usual practice-----------3  Previous bad experience from ID-----------------------------------4  Cannot afford to pay for health services--------------------------5  No transportation services-------6  Cannot pay for transportation services----------------------------7  My husband decision ------------8  Religious restriction---------9  Other reasons(specify)---------99 |  |
| 308 | Have you faced any health problems immediately after delivery of your last child? | No-----------------------0  Yes(specify)---------------99 |  |
| 309 | Who made the final decision about your place of last delivery? | Wife/me----------------------------0  Husband ---------------------------1  Joint discussion-------------------2  Other (specify) ------------------99 |  |
| 310 | Did you have Antenatal visits to health facility during your last delivery? | No-----------------------0  Yes___________visits |  |

**Now I have completed my questions thank you very for your cooperation!**
